# Intravascular Ultrasound-Guided Versus Angiography-Guided Percutaneous Coronary Intervention With Drug-Eluting Stents: A Meta-analysis of Randomized Trials

**DOI:** 10.1016/j.jscai.2023.101045

**Published:** 2023-06-03

**Authors:** Rohit Vyas, Mitra Patel, George V. Moukarbel, Rajesh Gupta

**Affiliations:** Division of Cardiovascular Medicine, University of Toledo, Toledo, Ohio

**Keywords:** cardiac death, intravascular ultrasound, meta-analysis, percutaneous coronary intervention

## Introduction

Percutaneous coronary intervention (PCI) with drug-eluting stents (DES) is widely used in the management of acute coronary syndromes and severe, symptomatic coronary artery disease. Several randomized controlled trials (RCTs) have shown reduction in the rates of definite or probable stent thrombosis, target lesion revascularization, and major adverse cardiovascular event (MACE) with intravascular ultrasound (IVUS)-guided compared with conventional angiography-guided PCI.^1^, ^2^, ^3^, ^4^, ^5^, ^6^, ^7^ The current American College of Cardiology/American Heart Association guidelines assign IVUS-guided PCI a class IIa recommendation. Similarly, a class IIa recommendation was designated by the European Society of Cardiology. Despite multiple RCTs and an increasing prevalence of complex coronary lesions, contemporary utilization of IVUS during PCI in the United States remains quite low at approximately 5.6%.^8^

Prior meta-analyses have evaluated the role and impact of IVUS-guidance in PCI; however, many of these studies included observational data or studies from the bare metal stent (BMS) era. With data from the recently published RENOVATE-COMPLEX-PCI RCT,^5^ we have now conducted the most up-to-date meta-analysis of only high quality RCTs of IVUS-guided vs angiography-guided PCI from the DES era.

## Methods

We used Preferred Reporting Items for Systematic Reviews and Meta-Analyses (PRISMA) guidelines to search databases for RCT studying IVUS-guided vs angiography-guided PCI that were performed in the DES era. We excluded observational data and studies performed in cohorts receiving BMS. The primary end point was MACE, which was a composite of cardiac death, myocardial infarction, and target lesion revascularization (TLR). Secondary outcomes included individual components of the primary composite outcome as well as definite or probable stent thrombosis. We used the random-effect model of DerSimonian and Laird to calculate the aggregated odds ratio (OR) and corresponding 95% CI for MACE, definite or probable stent thrombosis, TLR, and cardiac death. Heterogeneity was assessed using the Higgins *I*^*2*^ statistic, with values <25% and >75% considered indicative of low and high heterogeneity, respectively.

## Results

A total of 5847 patients from 7 RCT were included in this study. The average age was 64.3 years. Of the patients, 34% had diabetes mellitus (33% IVUS cohort, 35% angiography cohort), 67% had hypertension (67% IVUS cohort, 38% angiography cohort), and 52% had hyperlipidemia (50% IVUS cohort, 52% angiography cohort). In total, 53% of the entire cohort presented with acute coronary syndrome, 20% underwent chronic total occlusion intervention, and 19% presented with bifurcation lesions. Five out of 7 studies reported treated lesion lengths, and the average lesion length for these 5 studies was 31.5 mm.

**Figure 1:**
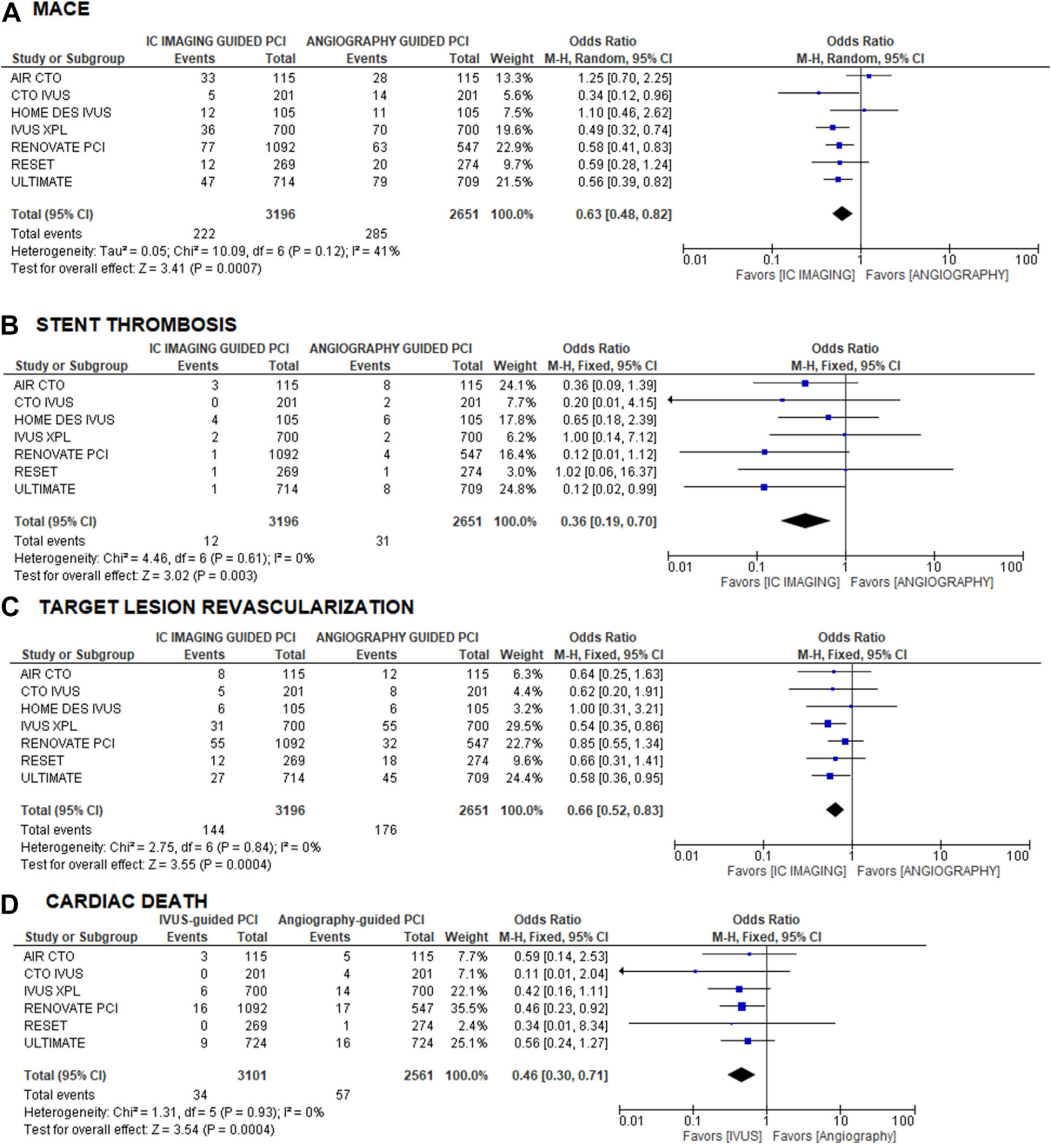
**Forest plots comparing IVUS-guided DES PCI vs angiography-guided DES PCI.** (**A**) MACE (primary composite outcome of cardiac death, target vessel MI, and TLR); (**B**) definite or probable stent thrombosis; (**C**) TLR; (**D**) cardiac death. DES, drug-eluting stents; IVUS, intravascular ultrasound; MACE, major adverse cardiovascular events; MI, myocardial infarction; PCI, percutaneous coronary intervention; TLR, target lesion revascularization.

The primary composite end point of MACE occurred in a significantly lower proportion of patients treated with IVUS-guided PCI compared with angiography-guided PCI (6.9% vs 10.7%; OR, 0.63; 95% CI, 0.48-0.82; *P* = .0007; *I*^*2*^ = 41%). IVUS-guided PCI was associated with lower rates of definite or probable stent thrombosis (0.4% vs 1.2%; OR, 0.36; 95% CI, 0.19-0.70; *P* = .003, *I*^*2*^ = 0%) and cardiac death (1.1% vs 2.2%; OR, 0.46; 95% CI, 0.3-0.71; *P* = .0004, *I*^*2*^ = 0%). Six of 7 studies reported rates of cardiac death. Lower rates of TLR were also seen with IVUS-guided PCI (4.5% vs 6.6%; OR, 0.66; 95% CI, 0.52-0.83; *P* = .0005, *I*^*2*^ = 0%). (Figure 1).

## Discussion

The results of our meta-analysis demonstrate significant reductions in MACE (37%), definite or probable stent thrombosis (64%), and TLR (34%) in patients treated with IVUS-guided PCI compared with angiography-guided PCI. In addition, there were significant differences in cardiac death, with rates of 1.1% with IVUS-guided PCI vs 2.2% with angiography-guided PCI. This represents a statistically significant 50% reduction in cardiac death. Importantly, cardiac death is a relatively rare end point, and meta-analysis is well-suited for determining efficacy for rare end points. Six of the studies in this meta-analysis reported cardiac death, for a total sample size of 5637 subjects, allowing for a robust determination of the effect of IVUS-guided PCI on cardiac death. These results provide important evidence of improved efficacy with IVUS-guided PCI and reflect the impact of protocolized interpretation of intracoronary imaging on several technical aspects of the PCI procedure, including choice of plaque modification techniques, stent sizing, deployment location, optimal stent length, and degree of postdilation for PCI optimization.

Our study included the 7 largest RCT conducted on IVUS-guided PCI, which we believe further increases the robustness of our findings. Furthermore, we elected to include RCTs performed in the DES era and excluded all RCTs performed in patients receiving BMS to provide an analysis that better reflects contemporary practice patterns. Finally, we included the recently published results of the RENOVATE-COMPLEX-PCI RCT.

One potential confounding factor in our study is that 26% of the patients in RENOVATE-COMPLEX-PCI underwent optical coherence tomography-guided DES implantation; however, this only represents 8% of the population that underwent imaging-guided PCI. Also, the 3 major RCT (IVUS-XPL,^4^ ULTIMATE,^7^ and RENOVATE PCI^5^) were performed in East Asian populations, which might limit the generalizability of the trial results to Western populations. However, these trials were all very well-designed and powered to detect clinically important outcomes. In addition, there is an ongoing US trial, IMPact on Revascularization Outcomes of IVUS-Guided Treatment of Complex Lesions and Economic Impact (IMPROVE) (NCT04221815). However, there have been some concerns about equipoise now that 3 major RCT have demonstrated consistent results, and this trial may have difficulty with enrollment.

The composite of these studies further reflects the fact that lesion complexity continues to increase; across all the studies, the average lesion length was 31.5 mm, 20% of the overall cohort presented with chronic total occlusions, and 22% of the patients in RENOVATE-COMPLEX-PCI and 24% of the patients in ULTIMATE underwent bifurcation PCI. In the context of the results of these studies and our meta-analysis results, it appears clear that imaging-guided PCI will provide patients with significantly improved outcomes. With multiple RCTs demonstrating the superior efficacy of IVUS-guided PCI, intracoronary imaging should receive a higher class of recommendation from cardiology and interventional cardiology professional societies. Furthermore, interventional cardiology training programs should emphasize the use of intracoronary imaging-guided PCI, and quality metrics should provide feedback on the percentage of PCI cases performed with intracoronary imaging guidance. Further educational efforts focused on implementing a simple, protocolized approach to using IVUS information for PCI optimization should also be pursued.

## Declaration of competing interest

The authors declared no potential conflicts of interest with respect to the research, authorship, and/or publication of this article.
